# Disseminated tegumentary leishmaniasis refractory to liposomal amphotericin B treatment

**DOI:** 10.1590/0037-8682-0655-2020

**Published:** 2021-03-08

**Authors:** Ana Flávia Borges, Juliana D'Andrea Molina, Marilda Aparecida Milanez Morgado de Abreu

**Affiliations:** 1 Hospital Regional de Presidente Prudente, Departamento de Clínica Médica, Presidente Prudente, SP, Brasil.; 2 Hospital Regional de Presidente Prudente, Departamento de Dermatologia, Presidente Prudente, SP, Brasil.

**FIGURE 1:**
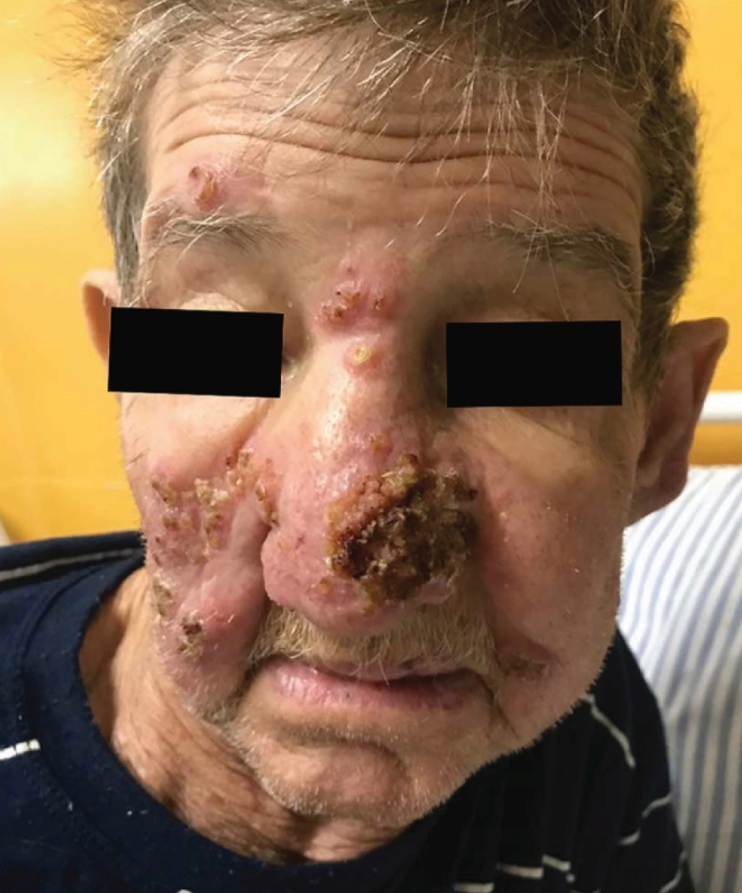
Erythematous, infiltrative, and exulcerated plaque covered with crusts on the face.

Present a unique case of atypical presentation of exuberant cutaneous leishmaniasis refractory to treatment. A 77-year-old man presented with infiltrative erythematous plaque with crusting in the right malar region, which progressed as diffuse skin lesions in the nasal, contralateral malar, and forehead regions (Figure 1). The lesion was subjected to biopsy, which confirmed the occurrence of tegumentary leishmaniasis.

He was referred to our service. Despite being treated with long-cycle of liposomal amphotericin B (AmB), the lesions continued to spread to the lower and upper limbs. A total accumulated dose of 40 mg/kg was administered to the patient using the Unified Health System (SUS).

**FIGURE 2:**
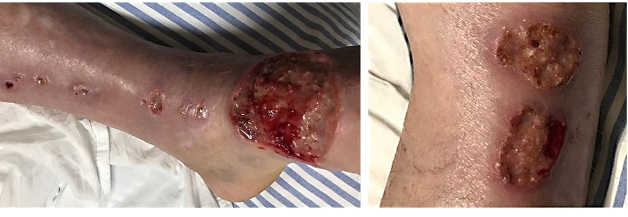
Multiple ulcers with elevated and infiltrative edges distributed on the lower left limb.

**FIGURE 3:**
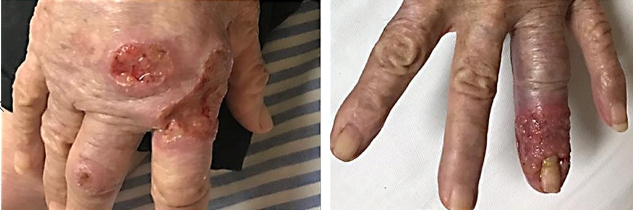
Multiple ulcers with elevated and infiltrative edges distributed on the upper limbs.

The patient exhibited signs of relapse with new skin lesions and an increase in the previous ones (Figure 2 and Figure 3). The medical team decided to administer miltefosine; however, it was not available in SUS. While miltefosine was being procured, the patient presented with decompensated heart failure and expired. The etiology of decompensation remains ambiguous; however, hemodynamic instability might have occurred due to high infusion volumes and renal injury.

Pentavalent antimonials and AmB are presently the drugs of choice for treating tegumentary leishmaniasis. However, AmB is prescribed in severe cases or when pentavalent antimonials are contraindicative. Both treatments have shown significantly reduced efficacy recently^1^.

There is limited therapeutic arsenal in the SUS and the possibility of drug resistance cannot be overlooked. The administration and availability of miltefosine can provide significant therapeutic benefits. The combination therapy may enable better adherence due to fewer side effects. Moreover, this drug can act synergistically to control leishmaniasis^2^.
